# Modulating innate immune activation states impacts the efficacy of specific Aβ immunotherapy

**DOI:** 10.1186/s13024-021-00453-4

**Published:** 2021-05-06

**Authors:** Yona Levites, Cory Funk, Xue Wang, Paramita Chakrabarty, Karen N. McFarland, Baxter Bramblett, Veronica O’Neal, Xufei Liu, Thomas Ladd, Max Robinson, Mariet Allen, Minerva M. Carrasquillo, Dennis Dickson, Pedro Cruz, Danny Ryu, Hong-Dong Li, Nathan D. Price, NIlüfer Ertekin-Taner, Todd E. Golde

**Affiliations:** 1 Department of Neuroscience and Neurology, Center for Translational Research in Neurodegenerative Disease, and McKnight Brain Institute, University of Florida, FL 32611 Gainesville, USA; 2 Institute for Systems Biology, WA 98109 Seattle, USA; 3 Department of Health Sciences Research, Mayo Clinic Florida, 32224 Jacksonville, FL USA; 4 Department of Neuroscience, Mayo Clinic, 32224 Jacksonville, FL USA; 5 Center for Bioinformatics, School of Computer Science and Engineering, Central South University, Hunan 410083 Changsha, People’s Republic of China; 6 Department of Neurology, Mayo Clinic, 32224 Jacksonville, FL USA

**Keywords:** Amyloid, Immunotherapy, Inflammation, Il6, Il10, IL6, IL10, Alzheimer’s disease, Adenoassociated virus, RNA seq

## Abstract

**Introduction:**

Passive immunotherapies targeting Aβ continue to be evaluated as Alzheimer’s disease (AD) therapeutics, but there remains debate over the mechanisms by which these immunotherapies work. Besides the amount of preexisting Aβ deposition and the type of deposit (compact or diffuse), there is little data concerning what factors, independent of those intrinsic to the antibody, might influence efficacy. Here we (i) explored how constitutive priming of the underlying innate activation states by Il10 and Il6 might influence passive Aβ immunotherapy and (ii) evaluated transcriptomic data generated in the AMP-AD initiative to inform how these two cytokines and their receptors’ mRNA levels are altered in human AD and an APP mouse model.

**Methods:**

rAAV2/1 encoding EGFP, Il6 or Il10 were delivered by somatic brain transgenesis to neonatal (P0) TgCRND8 APP mice. Then, at 2 months of age, the mice were treated bi-weekly with a high-affinity anti-Aβ1–16 mAb5 monoclonal antibody or control mouse IgG until 6 months of age. rAAV mediated transgene expression, amyloid accumulation, Aβ levels and gliosis were assessed. Extensive transcriptomic data was used to evaluate the mRNA expression levels of IL10 and IL6 and their receptors in the postmortem human AD temporal cortex and in the brains of TgCRND8 mice, the later at multiple ages.

**Results:**

Priming TgCRND8 mice with Il10 increases Aβ loads and blocks efficacy of subsequent mAb5 passive immunotherapy, whereas priming with Il6 priming reduces Aβ loads by itself and subsequent Aβ immunotherapy shows only a slightly additive effect. Transcriptomic data shows that (i) there are significant increases in the mRNA levels of Il6 and Il10 receptors in the TgCRND8 mouse model and temporal cortex of humans with AD and (ii) there is a great deal of variance in individual mouse brain and the human temporal cortex of these interleukins and their receptors.

**Conclusions:**

The underlying immune activation state can markedly affect the efficacy of passive Aβ immunotherapy. These results have important implications for ongoing human AD immunotherapy trials, as they indicate that underlying immune activation states within the brain, which may be highly variable, may influence the ability for passive immunotherapy to alter Aβ deposition.

## Introduction

Accumulation of Aβ aggregates in the brain parenchyma is hypothesized to trigger a complex neurodegenerative cascade that ultimately results in Alzheimer’s disease (AD). Based on this hypothesis there has been intense interest in therapeutic targeting of Aβ and Aβ aggregates [1–3]. Numerous immunotherapeutic approaches to targeting Aβ have been evaluated in preclinical rodent models of Aβ accumulation and multiple antibodies and active immunotherapies have advanced to clinical trials [4–10]. Indeed, preclinical studies have repeatedly established the disease-modifying potential of anti-Aβ immunotherapy. However, the results to date from many human anti-Aβ immunotherapy trials have been disappointing [11–16].

Data from the aducanumab phase 1b study suggested that reductions of CNS amyloid, or at least the amyloid ligand PET signal, can be achieved in relatively short time-periods [8, 17, 18]. Though initial phase 1b trial data suggested that this amyloid reduction might be associated with functional and cognitive benefits [8], the phase 3 trial targeting treatment in mild AD was initially halted due to lack of clinical efficacy [19]. However, more recent reanalysis of the trial with some additional data suggests clinical efficacy associated with the high dose treatment. These reanalyzed data, whose interpretation is controversial [20, 21], support a new biologic drug application that is currently being reviewed by the FDA. Similar reports of reduced amyloid PET ligand binding following immunotherapy have been reported with the antibody BAN2401 [19, 22], and phase 3 studies of this antibody in symptomatic AD are ongoing.

Even though amyloid ligand reduction binding has been observed, it is not yet clear how well this will correlate with alterations in amyloid levels in the postmortem brain. Further, it did not appear that everyone treated with these antibodies showed large reductions in the PET amyloid signal. Despite the lack of evidence for truly robust and universal efficacy in terms of slowing functional and cognitive decline, there is hope that Aβ immunotherapies, with evidence for target engagement in humans, especially if used in the preclinical stages of AD or in primary prevention, could still show meaningful clinical efficacy [23].

However, there are still significant gaps in our understanding regarding the mechanism of anti-Aβ immunotherapies to reduce Aβ deposition [24–29]. One of the original hypotheses regarding a peripheral sink induced by the high concentration of free antibody in the periphery has largely been ruled out [27, 30]. Indeed, even in humans, robust peripheral target engagement of soluble Aβ with the central domain monomer selective antibody, solanezumab, has failed to reduce amyloid plaques and was not associated with significant functional or cognitive benefit in mild AD [15, 31]. Both Fc-dependent and Fc-independent mechanisms have been proposed to underlie efficacy, and there is robust data generated using different anti-Aβ antibodies and different preclinical models to either support or refute either mechanism [32–35]. Fc-dependent mechanism are purported to result in microglial activation and subsequent clearance of deposited Aβ. Fc-independent mechanisms likely work by binding aggregates and possibly interfering with subsequent aggregation, or enhancing efflux of the bound Aβ engaged by the antibody in the brain to the periphery. One possible factor that may help to explain the different preclinical observations regarding Fc-activation is that diffuse Aβ deposits seem to be reduced more with antibodies that activate Fc receptors than do the more densely cored plaques [26, 35–37]. Notably, Fc-dependent microglial activation following plaque engagement is the mechanisms of action proposed for aducanumab and supported by preclinical data with the murine version of that human antibody [8].

We and others have previously shown that altering innate immune activation states in the mouse brain via expression of cytokines, exposure to LPS or genetic manipulations can alter the time course of amyloid deposition in APP transgenic mice. Our internal data is consistent, showing that immune activating anti-inflammatory cytokines decrease amyloid loads and immune inhibitory, anti-inflammatory cytokines increase amyloid [38–43]. Other data in the field creates a more confusing picture (reviewed in [44, 45]). Many published studies show similar data with pro-inflammatory manipulations, whereas other data show that activation of an anti-inflammatory state can reduce Aβ accumulation or knockout of immune activating protein results in more amyloid deposition [38, 46–50]. Fewer immune manipulations have been reported in tau mice [51–53]. The handful of published studies suggest that there may be opposite effects of immune manipulations on Aβ and tau pathology [54–56]. For example, manipulation of CX3CR1 and CX3Cl1 seem to have opposite effects on Aβ and tau pathologies [57].

One concern with all of the studies we collectively do in our mouse models of AD pathology is that they are kept in relatively sterile conditions (e.g., specific pathogen free housing), meaning that they are subject to limited immune priming [58, 59]. Herein, we explored how immune priming via constitutive expression of Il6 or Il10 influenced subsequent passive Aβ immunotherapy. We also evaluated how IL6 an IL10 and their receptors are altered at a transcriptomic level in (i) AD temporal cortex and cerebellar cortex in a large series of AD and Control brains and (ii) in longitudinal cohorts of APP TgCRND8 mice. We find that mAb5 passive immunotherapy alone and expression of rAAV-Il6 significantly attenuated Aβ accumulation, whereas expression of rAAV-Il10 significantly increased Aβ accumulation. rAAV-Il6 in combination with mAb5 resulted in a significant decrease in Thioflavin S positive plaque counts compared to either intervention alone, but the effect was only slightly additive. In contrast, rAAV-Il10 preconditioning completely abrogated the beneficial effect of mAb5 immunotherapy on amyloid deposition. Large-scale transcriptomic data reveal that Il10 and Il6 and their receptors show quite variable expression in the brain, in both humans and mouse models. These results have important implications for ongoing human AD immunotherapy trials, as they indicate that underlying immune activation within the brain may influence the ability of passive immunotherapy to alter Aβ deposition.

## Results

### Overexpression of rAAV-Il10 and Il6 in the brain induce robust gliosis

**Fig. 1:**
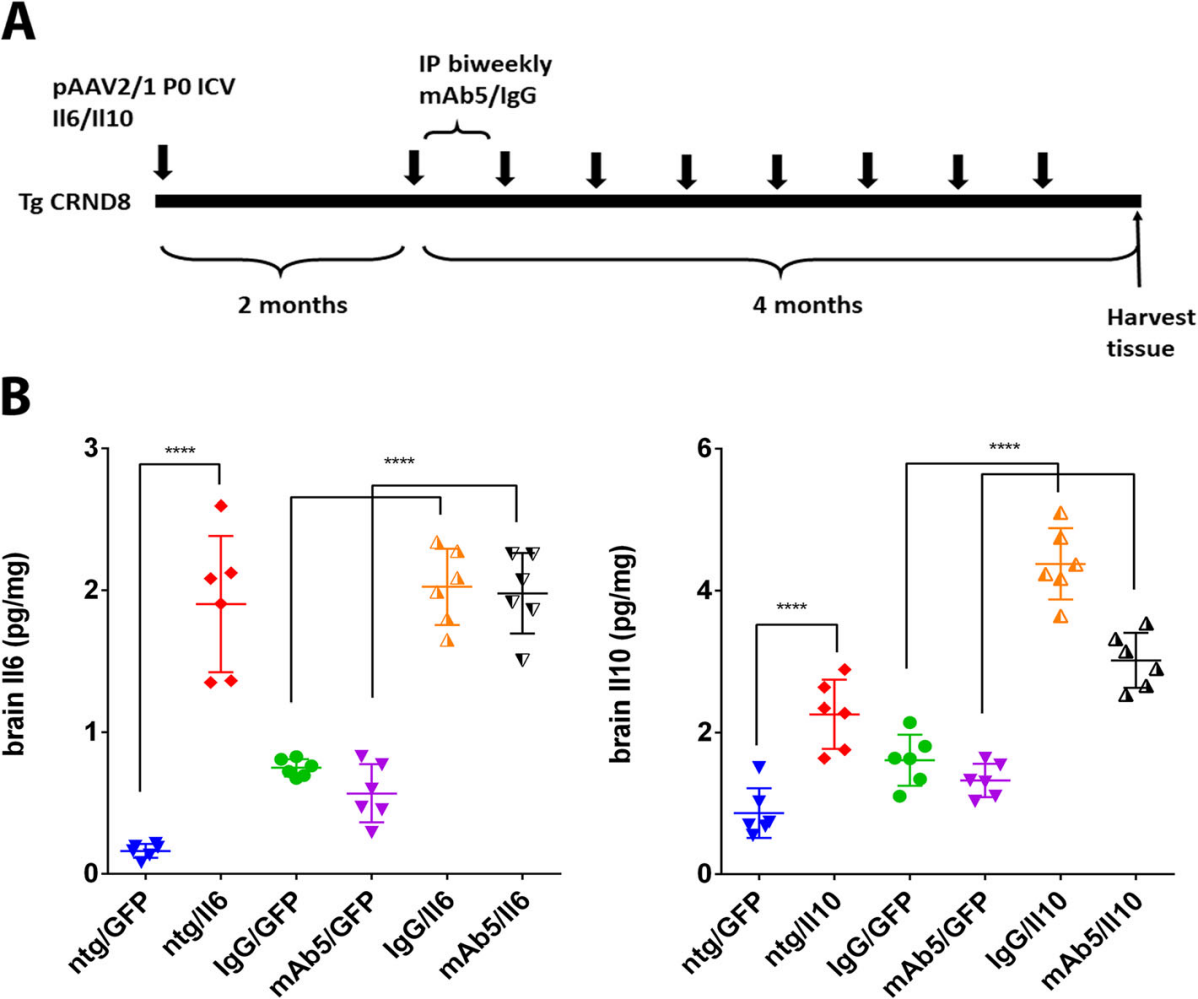
Il10 and Il6 levels in the brain and plasma. **a** Schematic representation of experimental paradigm. Neonatal TgCRND8 mice were bilaterally injected ICV with pAAV2/1 (4 × 10^10^ genomes) expressing Il6 or Il10 cytokines. Control mice were injected with pAAV2/1-GFP. At 2-months-old mice were divided into two groups that were immunized bi-weekly with 500 µg of mAb5 or mouse IgG. **b** 6-month-old mice were sacrificed and brains were harvested (*n* = 6–12). Brain levels of Il6 and Il10 were measured by ELISA. Data represent mean + SEM. *p < 0.01, **p < 0.001, ****p < 0.0001

**Fig. 2:**
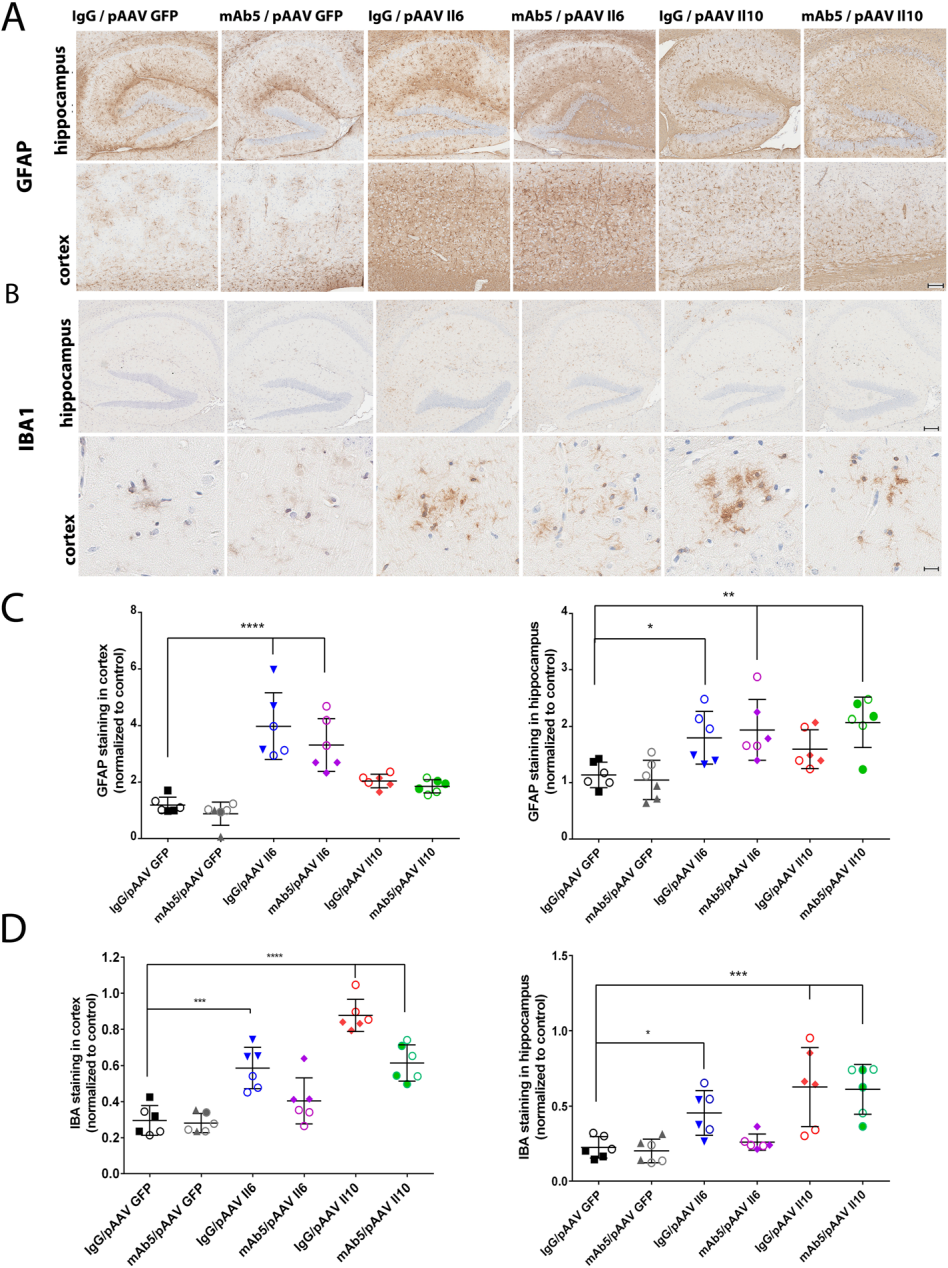
Increased gliosis and astrocytosis as a result of Il6 and Il10 overexpression combined with mAB5 immunotherapy. **a** Up-regulation of activated glia was determined by IBA1 immunoreactivity in hippocampal (top panels) and higher magnification sections of cortex (bottom panels) of Il6, Il10 and EGFP injected TgCRND8 mice immunized with anti-Aβ mAb5 or mouse IgG. Abundant activated microglia are present in Il6-injected mice compared with EGFP-expressing control mice, and even more so in Il10 injected mice. mAb5 immunization had no significant effect on amount of activated microglia. Scale bars = 150 μm (top); 45 μm (bottom). **b** Activated astrocytes in the cortex of Il6, Il10 and EGFP injected TgCRND8 mice immunized with anti-Aβ mAb5 or mouse IgG were detected by immuno staining of paraffin sections with rabbit polyclonal anti-GFAP antibody. Representative hippocampal sections (top panels) along with cortical sections (bottom panels) show a robust increase in the number of GFAP-positive neurons following Il6 expression. Scale bars = 150 μm. **c**, **d** Quantitative burden analysis of GFAP and IBA1 -positive cells in the cortex and hippocampus shows significant increase in Il6 and Il10 cohorts. The immunostaining was quantified from three sections from each mouse brain using Aperio imaging algorithms. Empty circles represent male mice, full symbols – females. n = 6, *p < 0.05, **p < 0.01, ***p < 0.001, ****p < 0.0001, one-way Anova multiple comparison test

A schematic diagram of the experimental design used is shown in Fig. 1a. Briefly, we preconditioned TgCRND8 mice to express either mIL6 or mIL10 by P0 injection with rAAV2/1 vectors encoding these transgenes and began biweekly immunization with mAb5, an anti-Aβ1–16 IgG2b antibody, at 2 months of age. Mice were euthanized at 6 months of age. We confirmed that brain levels of Il6 and Il10 were increased even in 6-month-old mice (Fig. 1b) and showed that mAb5 or IgG control antibody treatment did not significantly alter expression levels of either cytokine. In previous studies, we noted that Il6 and Il10 altered gliosis, here we again assessed gliosis in all cohorts of mice. Staining with an IBA1 antibody reveals increased microgliosis in Il6 overexpressing brain in the cortex and hippocampus with no noticeable differences between groups immunized with mAb5 or control IgG (Fig. 2). Il10 overexpression increased IBA1 in the brains of overexpressing mice, and caused a slight morphological change with cells showing more amoeboid morphologies, especially around plaques. Il6 caused substantial increase in the amount of GFAP reactive astrocytes in the hippocampus and cortex, whereas Il10 caused a small but not significant increase in the GFAP staining. Interestingly, GFAP staining in mice overexpressing Il10 and treated with mAb5 is elevated as compared to control. Subgroup analysis showed no differences in responses of male versus female.

### Effects of Il6, mAb5, or both in combination on Aβ deposition

**Fig. 3:**
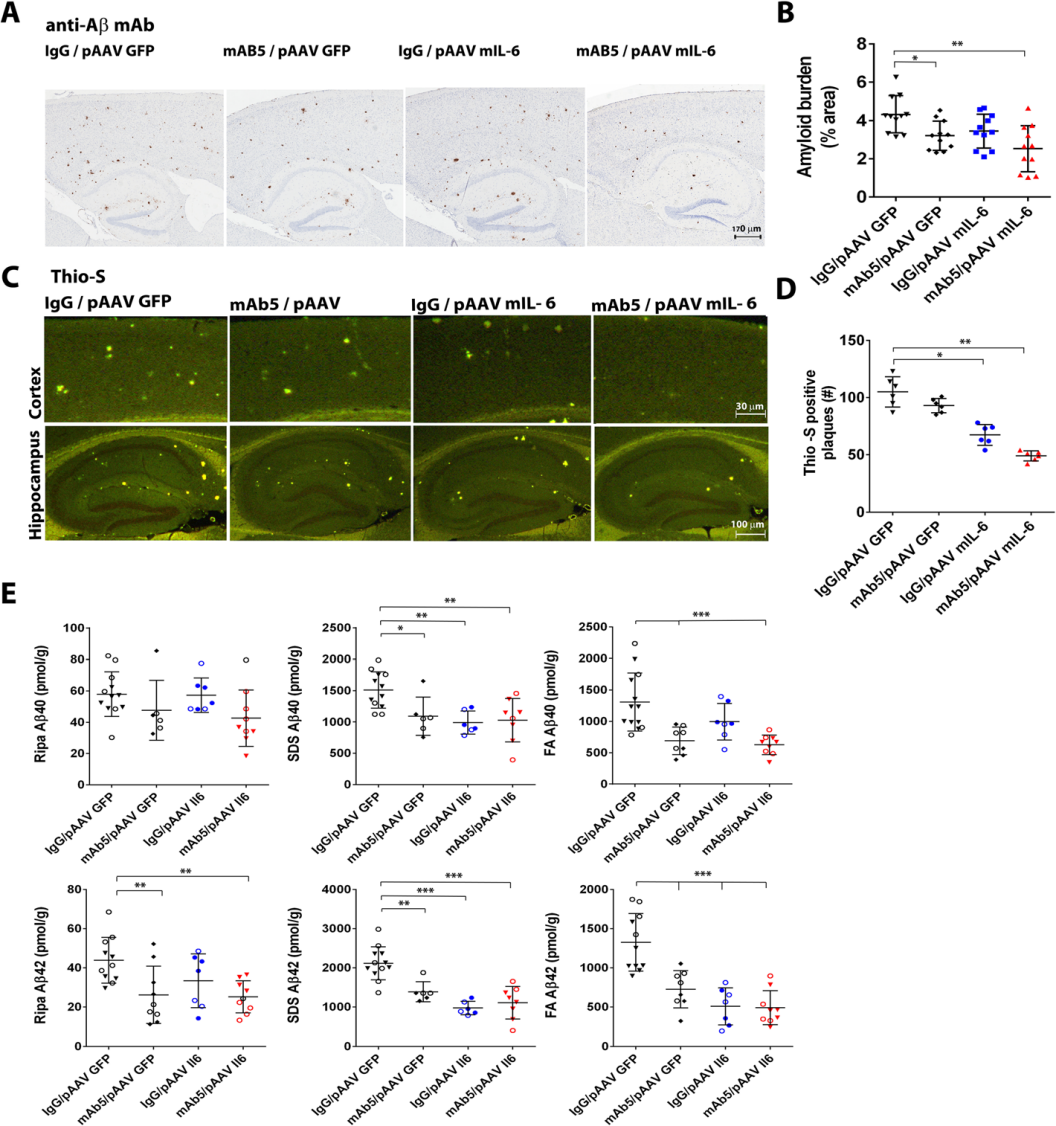
Effects of Il6 and Ab5 alone, or in combination on Aβ deposition. **a** Representative brain sections stained with pan-Aβ1–16 antibody (mAb 33.1.1) show Aβ plaque immunoreactivity in the cortex and hippocampus of 6-month-old TgCRND8 mice expressing Il6 or EGFP and immunized with mAb5 or mouse IgG. Scale bar = 150 μm. **b** The immunostaining was quantified from three sections from each mouse brain using Aperio imaging algorithms. Combination of Il6 overexpression and immunotherapy prevented plaque formation more, although not significantly, than each treatment individually. *n* = 5–7, *P < 0.05; **P < 0.01. **c** Representative fluorescent cortical and hippocampal sections stained with Thio-S show Aβ plaque immunoreactivity in the cortex and hippocampus of 6-month-old TgCRND8 mice expressing Il6 or EGFP and immunized with mAb5 or mouse IgG. Scale bars = 150 μm. **d** The number of Thio-S positive cored plaques was quantified from three sections from each mouse brain. Combination of Il6 overexpression and immunotherapy prevented plaque formation as well as Il6 overexpression alone. **e** Biochemical analyses of FA, SDS and RIPA extractable Aβ42 and Aβ40 levels in 6-month-old Il6-expressing TgCRND8 mice and EGFP-expressing age matched controls immunized with mAb5 or mouse IgG was performed by sandwich ELISA with anti Aβ40 and Aβ42 specific antibodies 2.13 and 13.1.1 as capture and 4G8-HRP as detection. Reduction in Aβ levels was achieved with both Il-6 overexpression and with immunotherapy, although a synergistic effect was not observed. Empty and full symbols represent male and female mice, respectively. *n* = 5–16, *p < 0.05, **p < 0.01, ***p < 0.001

Both mAb5 and Il6 reduce Aβ load, though the extent to which they alter Aβ accumulation depends on the methodology used to assess deposition. When immunohistochemical methods were used to assess overall Aβ deposition only the combination of mAb5 and Il6 showed a significant reduction (*p* < 0.01, One-way Anova with Turkey’s multiple comparison test) (Fig. 3a, b). When Thioflavin S was used to assess compact amyloid plaque number per section, there was a clear and significant reduction by Il6 alone and mAb5 in combination  with Il6 (*p* < 0.001 and *p* < 0.0001, respectively) (Fig. 3c, d). Notably, the combination of mAb5 and Il6 reduced plaque number to a greater extent as compared to either treatment alone (*p* < 0.0001 for mIL6 / mAb5 versus mAb5 alone and *p* < 0.01 versus Il6 alone). In neither of these histochemical assessments does the reduction in Aβ deposition by mAb5 reach statistical significance. Assessment of biochemical loads showed that mAb5 treatment alone reduced SDS-insoluble formic acid solubilized (FA) Aβ40 (*p* < 0.01) and Aβ42 (*p* < 0.001), RIPA-insoluble SDS soluble (SDS) Aβ40 (*p* < 0.05) and Aβ42 (*p* < 0.01), and RIPA-soluble (RIPA) Aβ42 (*p* < 0.1) (Fig. 3e). Il6 alone also reduced Aβ in these fractions compared to control as follows FA Aβ42 (*p* < 0.0001), SDS Aβ40 (*p* < 0.01) and Aβ42 (*p* < 0.0001), but did not have significant impact on RIPA soluble Aβ. The combination of mAb5 and Il6 also reduced Aβ compared to control: FA Aβ40 (*p* < 0.001) and Aβ42 (*p* < 0.0001), SDS Aβ40 (*p* < 0.01) and Aβ42 (*p* < 0.0001), and RIPA Aβ42 (*p* < 0.01). In contrast to the histochemical measures, the biochemical analysis do not show statistical evidence for increased reductions in Aβ loads with the combination of Il6 and mAb5 relative to either treatment alone.

### Effects of Il10, mAb5, or both in combination on Aβ deposition

**Fig. 4:**
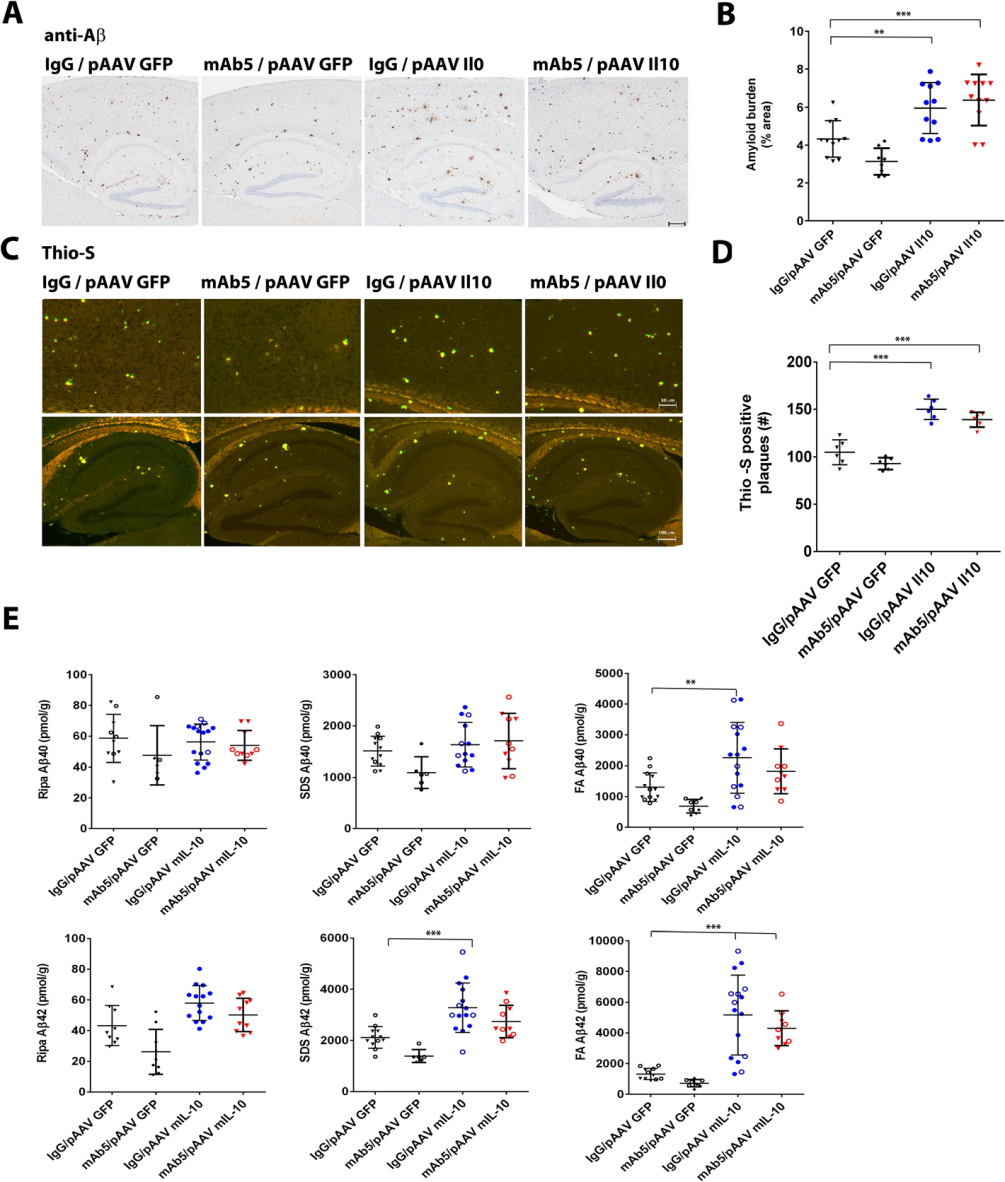
Effects of Il10 and mAb5 alone, or in combination on Aβ deposition. **a** Il10 abolishes the effect of immunotherapy on amyloid loads. Representative brain sections stained with pan-Aβ1–16 antibody (mAb 33.1.1) show Aβ plaque immunoreactivity in the cortex and hippocampus of 6-month-old TgCRND8 mice expressing Il10 or EGFP and immunized with mAb5 or mouse IgG. Scale bar = 150 μm. **b** The immunostaining was quantified from three sections from each mouse brain using Aperio imaging algorithms. Combination of Il10 overexpression and immunotherapy resulted in increased plaque formation. *n* = 5–16, *P < 0.05; **P < 0.01. **c** Overexpression causes an increase in number of Thio-S positive plaques. Il10 overexpression abolished the effects of mAb5 immunotherapy on reduction in the number of Thio-S positive plaques. **d** Representative fluorescent cortical and hippocampal sections stained Thio-S show Aβ plaque immunoreactivity in the cortex and hippocampus of 6-month-old TgCRND8 mice expressing Il10 or EGFP and immunized with mAb5 or mouse IgG. Scale bars = 150 μm. B. The number of Thio S positive cored plaques was quantified from three sections from each mouse brain. **e** Il10 overexpression-induced increase in Aβ levels is observed in in cohorts immunized with either mAb5 or mouse IgG as demonstrated by sandwich ELISA with anti Aβ40 and 42 specific antibodies 2.13 and 13.1.1 as capture and 4G8-HRP as detection. Immunotherapy had no effect on Aβ levels in Il10 overexpressing mice. *n* = 5–16, *p < 0.05, **p < 0.01, ***p < 0.001. Empty and full circles represent male and female mice, respectively

Overall, Il10 increased Aβ loads and prevented the ability of mAb5 to reduce Aβ. For these studies, the effects of Il10 on immunotherapy were evaluated and compared to the same rAAV-EGFP control group and mAb5 treatment group. Il10 markedly increased immunohistochemical loads both in the absence and presence of mAb5 (*p* < 0.05 for Il10 vs. GFP, and *p* < 0.01 for Il10/AB5 vs. GFP) (Fig. 4a, b). There was no statistical difference in the increase in Aβ load, as detected by IHC, between the Il10 alone group and the mAb5/Il10 group. When Thioflavin S was used to assess compact amyloid plaque number per section, there was a clear and significant increase by Il10 alone and mAb5 with Il10 combination (*p* < 0.0001 for both), and there was no statistical difference in the increase in Aβ between the Il10 treated group and the Il10 plus mAb5 treatment group (Fig. 4c, d). Assessment of biochemical loads showed that Il10 treatment alone increased FA Aβ40 (*p* < 0.05) and Aβ42 (*p* < 0.0001), SDS Aβ42 (*p* < 0.001), and RIPA Aβ42 (*p* < 0.05) (Fig. 4e). The combination of mAb5 and Il10 also increased FA Aβ42 (*p* < 0.01) and SDS Aβ42 (*p* < 0.01) compared to control. Notably, there was no statistical difference in any of the fractions between the Il10 alone group and the Il10 plus mAb5 group.

### Transcriptomic analysis of interleukins and their receptors

It is well established that mouse models of amyloid deposition and human AD brain show large scale transcriptomic and pathologic alterations in immune pathways [60]. Here we have specifically mined data regarding RNAseq based mRNA levels of these interleukins and their receptors that was generated by our consortium (https://adknowledgeportal.synapse.org/Explore/Projects?Grant%20Number=U01AG046139) within the larger AMP-AD: Accelerating Medicines Partnership - Alzheimer’s Disease Target Discovery and Preclinical Validation project (https://adknowledgeportal.synapse.org/Explore/Programs?Program=AMP-AD). Notably, for both the human and mouse studies we had used high read depth (> 100 M reads) to increase our ability to capture changes in low abundance mRNAs.

**Fig. 5:**
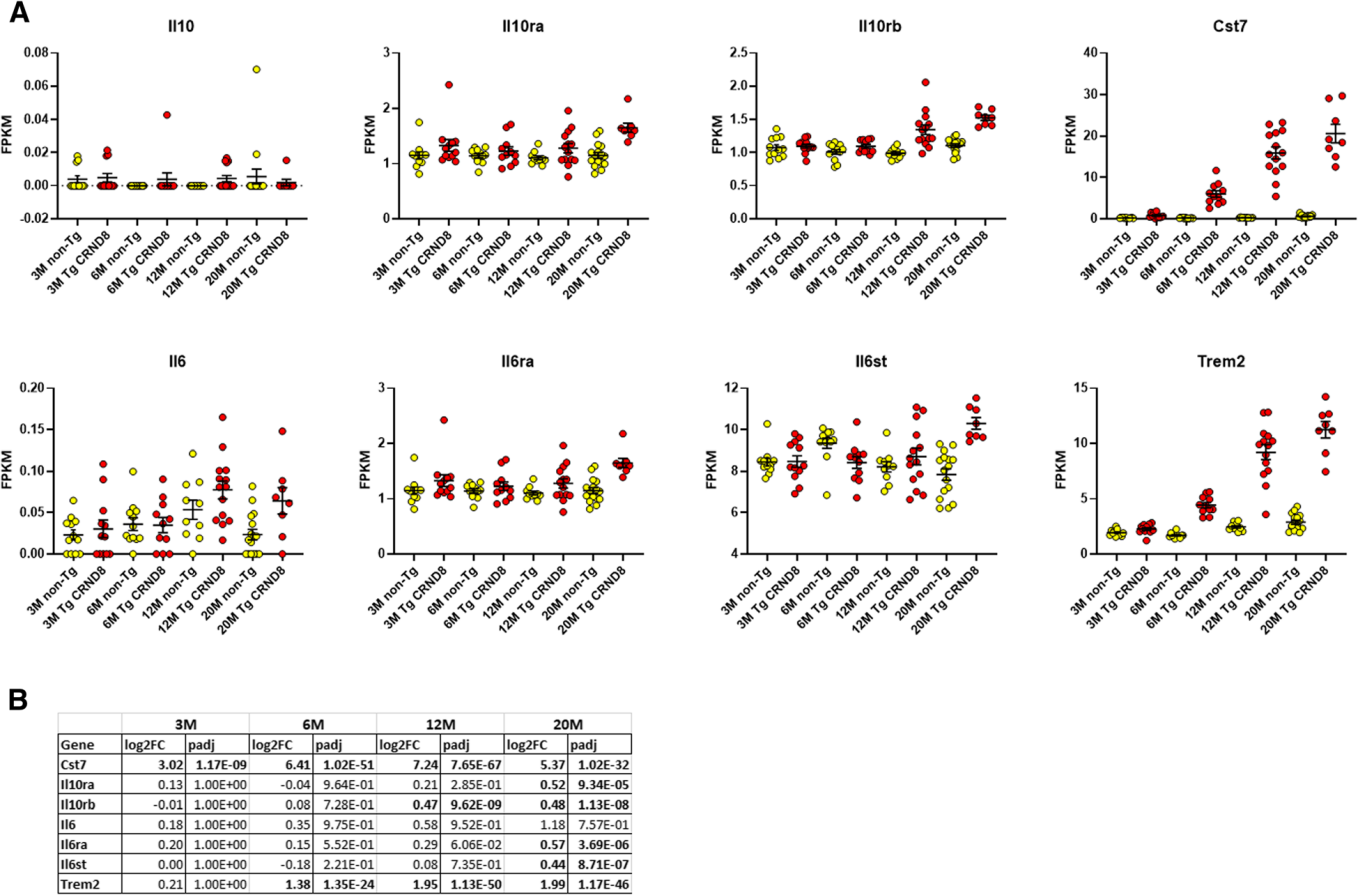
CRND8 mice show increased expression of Il6ra, Il6st, Il10ra, Il10rb, Trem2 and Cst7 mRNAs as they age. **a** RNAseq of the forebrain from 3, 6, 12 and 20-Month-old TgCRND8 mice and age matched littermate non-Tg mice. Each scatterplot shows data for the Fpkm values of the Tg and non-Tg at each age for the mRNA indicated. All the data underlying these analyses, detailed methods and the analyses themselves is found at https://adknowledgeportal.synapse.org/Explore/Studies?Study=syn3157182. **b** Summary statistics in terms of log 2 fold change (log2FC) (TgCRND8 vs. non-Tg) and adjusted p-values (padj) are shown for each age

**Fig. 6:**
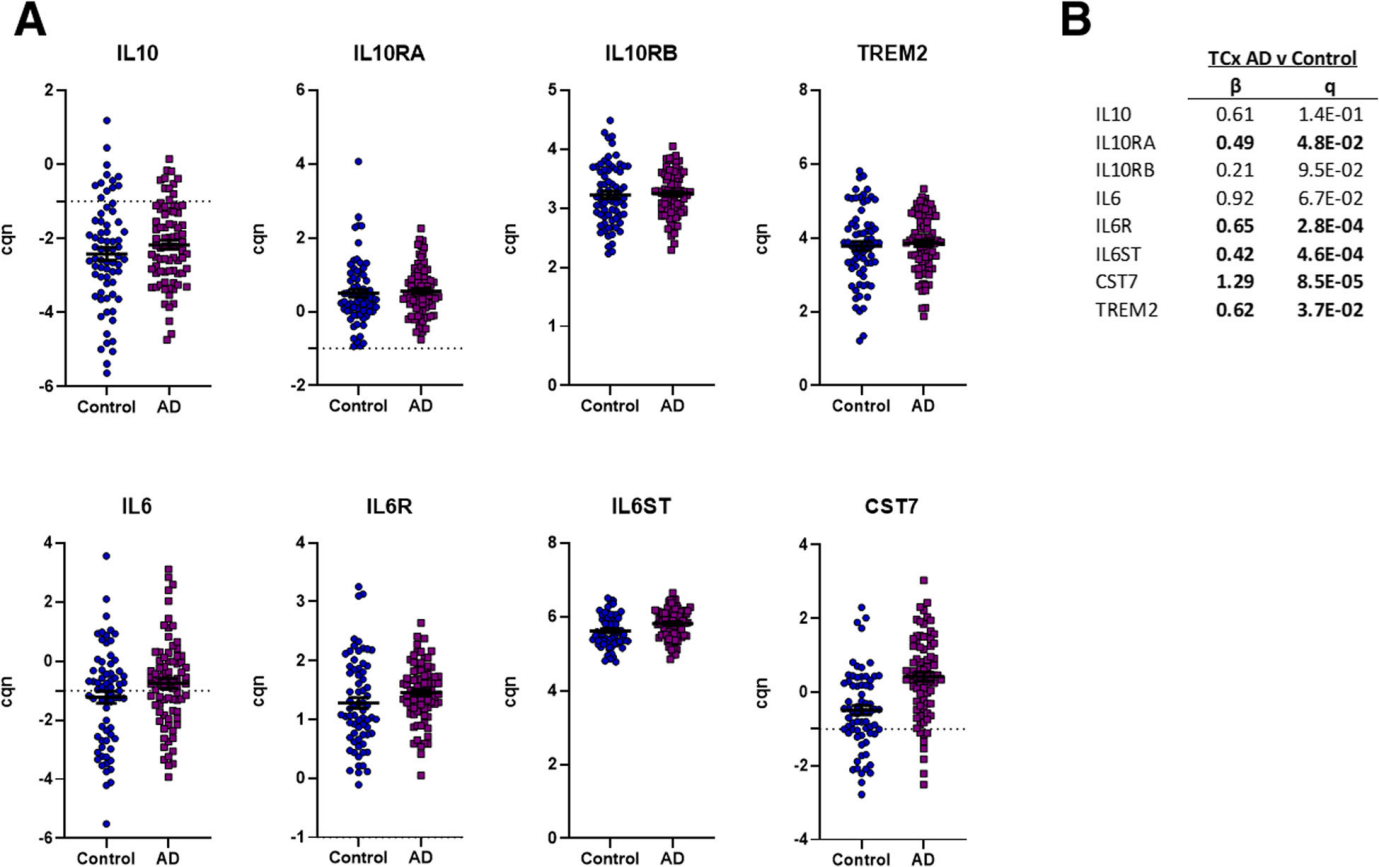
The AD temporal cortex has increased expression of IL6R, IL6ST, L10RA, TREM2 and CST7. **a** RNAseq of the Postmortem Temporal Cortex 69 control and AD brains. Each scatterplot shows data for the cqn values for the mRNA indicated. All the metadata underlying these analyses, detailed methods and the analyses themselves is found at https://adknowledgeportal.synapse.org/Explore/Studies?Study=syn5550404. Specific files relevant to this study are https://www.synapse.org/#!Synapse:syn6090811, https://www.synapse.org/#!Synapse:syn6122276, and https://www.synapse.org/#!Synapse:syn9786290. **b** Summary statistics in terms of β (AD v Control) and q values are shown

First, we examined mRNA expression of Il10, Il6, Il10ra (receptor subunit α), Il10rb (receptor subunit β), Il6ra (receptor subunit α), and Il6st (signal transducer) in TgCRND8 mice (APP KM670/671NL (Swedish), APP V717F (Indiana) compared to non-transgenic (non-Tg) mice at 3, 6, 12, and 20 months of age. For reference we also include data on Cst7 and TREM2 as two of the more significantly upregulated immune DEGs in the TgCRND8 mice. These data reveal that both Il10 and Il6 are expressed at very low levels in the mouse brain. Despite average read depths of over 100 million counts, Il10 mRNA is only detected in a few mice, and when it is detected the level is very low (< 0.1 FPKM). Il6 is detectable in a majority of the samples, but again all FPKM values are low (FPKM < 0.2). In contrast to the levels of the cytokines themselves, their receptors are expressed at appreciable levels and show upregulation in the aging TgCRND8 mice brains. At 20 month of age Il10ra, Il10rb, Il6ra and Il6st show significantly increased expression compared to non-Tg mice, with Il10rb also showing significant upregulation in 12month TgCRND8 mice brains (Fig. 5). Notably, this upregulation at the mRNA level is not as dramatic as that of other genes that are known to be quite selectively expressed on microglial cells. Indeed, many mRNAs, illustrated by Trem2 and Cst7 are markedly and significantly, upregulated at early ages in the TgCRND8 mice brains, and continue to increase in levels as the mice age. For the human data, we focused on a set of 80 AD and 69 control temporal cortex samples that had passed our RNAseq quality control. IL10 and IL6 were again generally expressed at low levels. However, in this case a number of the samples (~ 15 %) showed expression of IL10 and IL6 (~ 55 %) at levels higher than a cqn of -1. Both IL10 (p = 0.06) and IL6 (p = 0.02) showed trends towards increased in AD but these changes did not withstand correction for false discovery. IL10RA, IL6R, and IL6ST mRNA levels were all significantly increased in the AD temporal cortex, as were CST7 and TREM2 mRNA levels (Fig. 6).

## Discussion

We have found that preconditioning with Il6 or Il10 dramatically alters the effects of subsequent passive Aβ immunotherapy with the anti-Aβ antibody mAb5. Although a modest additive effect on one measure of Aβ deposition, (decreased Thioflavin S plaque count) was observed with Il6 preconditioning and subsequent mAb5 immunotherapy, Il10 preconditioning blocked the subsequent impact of mAb5 immunotherapy. The data has a high degree of validity as each of the manipulations by themselves replicated findings from our previous work [30, 38, 41]. Il10 expression increased Aβ deposition and astrocytosis. Il6 expression decreased Aβ deposition and produced both an astrocytosis and microgliosis. mAb5 immunotherapy alone decreased Aβ deposition to some degree without appreciable effect on astrocytes or microglial cells. In our previous studies, we had not performed a simultaneous comparison of the effect of Il6 and mAb5 immunotherapy, though our impression from those studies was that Il6 reduced Aβ almost as well as a passive Aβ immunotherapy [30, 41]. Here, we confirm that impression, and note that both treatments reduce Aβ deposition nearly to the same degree. The combination of Il6 and mAb5 shows no evidence for being synergistic and only appears to be partially additive, as the only significant impact of both Il6 and mAb5 together is on the number of Thioflavin S positive cored plaques.

There are a few caveats and limitations to this study. First, we have only evaluated the effects of preconditioning on the subsequent efficacy of a single anti-Aβ mAb. It is possible that other anti-Aβ mAbs, may be influenced to a greater or lesser degree by the immune preconditioning. Second, we have not looked at multiple time points. As we have previously shown that increased plaque load at time of immunization reduces efficacy of that therapy by itself, we would not expect that longer studies would appreciably alter the findings with Il10 preconditioning. It is, however, possible that longer treatment with both Il6 and mAb5 might show more robust effects of the combination. Third, we powered these studies to be able to detect effects on Aβ deposition, and the group sizes were not sufficient to evaluate behavioral impacts.

We have previously shown that suppression of an inducible mutant APP transgene in combination with passive anti-Aβ immunotherapy results in true clearance, not just suppression of ongoing deposition, of both the more diffuse Aβ deposits surrounding cored plaques as well as smaller non-dense core plaques. A similar study in the same inducible APP model, again shows that Il6 had a similar effect [61]. These data along with our data on Il6 preconditioning with subsequent mAb5 immunotherapy suggest that at least partially additive effects on Aβ accumulation and possibly clearance can be achieved through combinations of blocking Aβ production, aggregation and clearance.

Il10 preconditioning may abrogate the effect of subsequent Aβ immunotherapy through several non-exclusive mechanisms. Given our previous work and that of others showing the reduced efficacy of immunotherapy in mice with higher amyloid loads, it may simply be that Il10 increases Aβ deposition to the point that subsequent mAb5 administration is no longer effective [24, 58, 62–64]. We have also catalogued a large number of transcriptomic changes and functional alterations in microglial cells and microglial phagocytosis attributable to Il10 brain overexpression. Such complex changes induced by Il10 may also contribute to the lack of efficacy of subsequent passive immunotherapy.

The efficacy of immune checkpoint inhibitors in cancer is clearly influenced by the local tumor immune microenvironment [65] Herein, we have utilized publicly available transcriptomic data that we have generated to explore the notion that changes and variability in the immune system in individual human or mice brains might be an extrinsic factor that could alter responses to an anti-Aβ targeted immunotherapy. Taking a simplistic approach to this issue, we focused on how the cytokines studied herein and their receptors are altered both temporally in a mouse model of amyloid Aβ deposition and by the AD state. First, these data show that there are significant increases in the cytokine receptors mRNA levels in the TgCRND8 mouse model and in the temporal cortex of humans with AD. However, these receptor RNAs are not as robustly upregulated as much as some other microglial genes (e.g. TREM2, CST7). Second, these data show that in the mouse brain mRNAs for Il10, which is almost undetectable, and Il6 are expressed at much lower levels on average than their receptors. In human temporal cortex the difference between level of cytokine and level of receptor is not as large, but still appreciable. The relatively low levels of these cytokines mRNAs compared to their receptor mRNAs, prompt a number of intriguing questions that will need to be pursued in future studies. These include: Are these differences reflected at the protein level? How can such typically low levels of cytokines robustly engage the receptor? Is the periphery, typically, a primary or at least significant source of Il6 and Il10 in the brain? Third, and perhaps most striking, is that there is a great deal of variance in individual mouse brains and even more so in the human temporal cortex with respect to the relationship between the expression of IL6 and IL10 and the high affinity receptors IL6R and Il10RA. We believe this later feature is highly relevant to our experimental studies. Such data shows that in the individual human control or AD brain, there is a high degree of variability in the activation state of the immune system. Given our current data that Il10 and Il6 differentially alter the subsequent effects of anti-Aβ immunotherapy, we hypothesize that variation in innate immune activation states within the human brain, may contribute to the variability in response to an Aβ targeting immunotherapy, at least with respect to the effects on clearance of Aβ.

Of course, by themselves the variable levels of these select cytokines and receptors do not necessarily inform on the functional status of the immune system within the brain. Additional systems level multiomic studies including single cell studies and more comprehensive algorithms to predict immune status may help illuminate a set of biomarkers that better define, at an individual level, the brain’s immune status [66–68]. Ex vivo analysis of the immune microenvironment within a tumor has demonstrated utility in understanding how well immune checkpoint inhibitors may work. The lack of direct access to brain tissue in the AD field makes it much more challenging to assess immune status in an individual AD brain. Additional CSF and imaging biomarkers that better track innate immune status will be needed to better understand the influence of innate immune status on outcomes of anti-Aβ immune therapy [69].

In summary, our experimental data show that altering the brain’s immune activation state by priming with cytokines that have different effects by themselves on Aβ deposition can markedly impact the efficacy of subsequent passive anti-Aβ immunotherapy. These results have important implications for ongoing human AD immunotherapy trials, as they indicate that underlying immune activation states within the brain, which at least in the postmortem brain appear to be highly variable, may influence the ability of passive immunotherapy to alter Aβ deposition.

## Methods

### Animal models and AAV injection

**Mice.** All animal husbandry procedures performed were approved by the Institutional Animal Care and Use Committee. TgCRND8 were maintained as described before [70], transgenic males were crossed with B6C3F1 ntg females.

**rAAV2/1 viruses for ICV injections** expressing Il6 and Il10 under the control of the cytomegalovirus enhancer/chicken β actin promoter were generated as described previously [41]. Briefly, AAV vectors expressing the cytokines under the control of the cytomegalovirus enhancer/chicken beta actin (CBA) promoter, a WPRE, and the bovine growth hormone polyA were generated by plasmid transfection with helper plasmids in HEK293T cells. 48 h after transfection cells were harvested and lysed in the presence of 0.5 % Sodium Deoxycholate and 50U/ml Benzonase (Sigma) by freeze thawing, and the virus isolated using a discontinuous Iodixanol gradient, and affinity purified on a HiTrap HQ column (Amersham). The genomic titer of each virus was determined by quantitative PCR.

**Neonatal rAAV injections and antibody treatment.** TgCRND8 mice were injected with 2 µl of rAAV ICV into the both hemispheres using a 10 µl Hamilton syringe with a 30 g needle on day P0 (Il10) or P1 (Il6) as described before [38, 41] and aged till 2 months. They were then divided into two gender-matched cohorts, and immunized bi-weekly i.p. with mAb5 (IgG2b) or mouse IgG (0.5 mg/per mouse) diluted in 0.9 % saline, for 4 months, a regimen that was established in [30] .

**Measurement of Il6 and Il10 in the brain and plasma**. Brains from mice injected with rAAV Il6 and Il10 were sagitally dissected and the left hemisphere was snap-frozen in isopentane. They were then homogenized at a concentration of 150 mg/ml and sequentially extracted with protease inhibitor cocktail (Roche) in RIPA buffer, 2 % SDS buffer, and 70 % formic acid (FA) as described previously.

Sandwich capture Il6 ELISA assays using RIPA soluble lysates were done with mouse specific reagents (BD Biosciences). The  same procedure was performed on plasma from injected mice.

Aβ levels from the 2 % SDS– and 70 % FA–extracted samples were quantified using end-specific sandwich ELISA as previously described [71]. Aβ40 was captured with mAb 13.1.1 (human Aβ35–40 specific; T.E. Golde) and detected by HRP-conjugated mAb 33.1.1 (human Aβ1–16; T.E. Golde). Aβ42 was captured with mAb 2.1.3 (human Aβ35–42 specific; T.E. Golde) and detected by HRP-conjugated mAb 33.1.1 (human Aβ1–16; T.E. Golde). ELISA results were analyzed using SoftMax Pro software.

**Immunohistochemical imaging and image processing**. Right hemibrain was fixed in 4 % paraformaldehyde. Immunohistochemical staining was done using pan Aβ antibody 33.1.1 (1:1500, T. Golde), Iba-1 (1:1000; Wako), GFAP (1:500; Chemicon). 1 % Thioflavin S (Sigma) staining was done on paraffin embedded brain sections using established protocols. Immunohistochemically and fluorescent stained sections were captured using the Aperio Scanscope XT or FL image scanner and analyzed using either Aperio positive pixel count or ImageJ program. Brightness and contrast alterations were applied identically on captured images using Adobe Photoshop CS3.

**Quantification of Aβ deposition and gliosis.** Immunohistochemically and fluorescent stained sections were captured using the Scanscope XT or FL image scanner (Aperio) and analyzed using ImageScope program. Aβ plaque burden and intensity of astrogliosis staining was calculated using the Positive Pixel Count program (Aperio). At least three sections per sample, 30 μm apart, were averaged by a blinded observer to calculate plaque burden. For Thioflavin S quantitation, one section per sample was used by a blinded observer to manually count the plaques using Adobe Photoshop CS5.

**Statistical Analysis**. Data were analyzed using Prism 6 (GraphPad) and presented as mean ± SEM. Overall data were tested for normality and, after being deemed to have a normal distribution, were analyzed via one-way ANOVA followed by Dunnett’s multiple comparison test. All comparisons were done between various groups and control. Sex differences in TgCRND8 mice were assessed by a post hoc analysis of the cohorts. Final images were created using Photoshop CS5 (Adobe).

**RNaseq data.** RNA sequencing data for TgCRND8 transgenic mice was downloaded from Synapse (doi: 10.7303/syn3157182). The gene count matrix was normalized for sex, and sequencing batch using robust linear regression (lqs method, MASS package in R) after filtering genes with less than 1 CPM in at least 50 % of the samples, zero imputation and standardizing the covariates at their median value. Further details using this approach are described by Glusman et al. [72]. The normalized count matrix was used as input for analysis using DESeq2 [73]. Human RNA sequencing data was downloaded from Synapse (doi: 10.7303/syn5550404). Data for the moue studies is reported as FPKM with and for the human studies as cqn.

## Data Availability

All data generated or analyzed during this study are included in this published article [and its supplementary information files]. Data that was generated by our consortium (https://adknowledgeportal.synapse.org/Explore/Projects?Grant%20Number=U01AG046139) within the larger AMP-AD: Accelerating Medicines Partnership - Alzheimer’s Disease Target Discovery and Preclinical Validation project (https://adknowledgeportal.synapse.org/Explore/Programs?Program=AMP-AD).
